# Phenotypic Detection of Carbapenemase and AmpC-β-Lactamase Production among Extended Spectrum β-Lactamase (ESBL)-Producing *Escherichia coli* and *Klebsiella* spp. Isolated from Clinical Specimens

**DOI:** 10.3390/antibiotics13010031

**Published:** 2023-12-28

**Authors:** Zakaria Garba, Bérenger Kaboré, Isidore J. O. Bonkoungou, Magloire H. Natama, Toussaint Rouamba, Kaisa Haukka, Juha P. Kirveskari, Halidou Tinto, Lassana Sangaré, Nicolas Barro, Anu Kantele

**Affiliations:** 1 Department of Biochemistry and Microbiology, Université Joseph KI-ZERBO, Ouagadougou 03 BP 7021, Burkina Faso; isidore.bonkoungou@ujkz.bf (I.J.O.B.); nicolas.barro@ujkz.bf (N.B.); 2 Clinical Research Unit of Nanoro, Institut de Recherche en Sciences de la Santé, Ouagadougou 11 BP 218, Burkina Faso; berenger.kabore@crun.bf (B.K.); magloire.natama@crun.bf (M.H.N.); toussaint.rouamba@crun.bf (T.R.); tintoh@crun.bf (H.T.); 3 Department of Microbiology, University of Helsinki, 00014 Helsinki, Finland; kaisa.haukka@helsinki.fi; 4 Human Microbiome Research Program, Medical Faculty, University of Helsinki, 00014 Helsinki, Finland; 5 Helsinki Innovation Services Ltd., University of Helsinki, 00014 Helsinki, Finland; juha.kirveskari@helsinki.fi; 6 Department of Health Sciences, Université Joseph KI-ZERBO, Ouagadougou 03 BP 7021, Burkina Faso; lassana.sangare@ujkz.bf; 7 Meilahti Infectious Diseases and Vaccine Research Center MeiVac, Helsinki University Hospital, 00029 Helsinki, Finland

**Keywords:** ESBL, carbapenemase, AmpC-β-lactamase, *E. coli*, *Klebsiella* spp., hospitals

## Abstract

**Introduction**: Data on antimicrobial resistance (AMR) are sparse across numerous African countries, as microbiological analyses are not routinely conducted and surveillance data are not collected. Accordingly, clinical samples are not routinely tested for carbapenem-resistant bacteria and, therefore, the general understanding of their prevalence in the region remains limited. **Methods**: Between January 2020 and June 2022, we collected extended spectrum β-lactamase (ESBL)-producing Enterobacterales (ESBL-PE) isolates from five hospitals in Burkina Faso. After an initial culture on ESBL-selective media, the species were identified using API20E and isolates were tested against 13 antimicrobial agents using the disc diffusion method on Mueller–Hinton (MH) agar. ESBL production was confirmed via a double-disc synergy test. Production of carbapenemases and AmpC-β-lactamases and phenotypic co-resistance were determined. **Results**: Among the 473 ESBL-PE, 356 were ESBL-*E. coli* (ESBL-Ec) and 117 were *Klebsiella* spp. (ESBL-K). Of these isolates, 5.3% were carbapenemase and 5.3% were AmpC-β-lactamase-positive. Three types of carbapenemases were identified: 19 NDM, 3 OXA-48-like and 1 VIM. Two isolates produced both NDM and OXA-48-like carbapenemases. Carbapenemase producers were detected at all levels of healthcare. Co-resistance rates were up to 85% for aminoglycosides, 90% for sulfonamides, 95% for fluoroquinolones and 25% for chloramphenicol. Fosfomycin resistance was 6% for ESBL-Ec and 49% for ESBL-K (49%). **Conclusions**: Some of the ESBL-Ec and ESBL-K co-produced carbapenemases and/or AmpC-β-lactamases at all healthcare levels and in various sample types with high co-resistance rates to non-betalactams. Carbapenem resistance is no longer rare, calling for testing in routine diagnostics, a comprehensive resistance surveillance system and infection control within healthcare.

## 1. Introduction

The emergence and spread of multidrug-resistant (MDR) bacteria are serious global public health threats. In 2019, an estimated number of 4.95 million deaths were associated with antimicrobial resistance (AMR), with 1.27 million directly attributable to MDR bacteria [1]. The highest burden is in Western sub-Saharan Africa, with 27.3/100,000 AMR-attributable and 114.8/100,000 AMR-associated deaths [1]; the same region where AMR surveillance is minimal and resistance data are limited. The healthcare costs of AMR reach nearly USD 1.2 trillion in the “High-AMR” case [2]. According to the World Bank estimation/projection, by 2030, there will be an annual increase of USD 0.22 trillion in the extra health expenditure incurring in low-AMR settings [2].

Carbapenem resistance among *Enterobacterales* has experienced a dramatic increase and global spread over the past decade, with reports both from hospital and community settings [3]. The emergence of carbapenem resistance substantially limits the therapeutic options at hospitals worldwide since carbapenems are last-resort antibiotics [3,4], and, in accordance, infections due to carbapenem-resistant *Enterobacterales* (CRE) are associated with increased mortality rates [5,6,7]. Management of patients with CRE infections often requires combination therapy, which involves various types of antibiotics, such as tigecycline and colistin [8,9]. Microbiological analyses are a prerequisite for accurate treatment of patients with CRE infections. However, in low- and middle-income countries (LMICs), resistance data are scarce due to the challenges in detecting carbapenemases in microbiology laboratories [10,11]. Special attention should also be paid to ESBL-producing bacteria, which co-produce carbapenemases and/or AmpC, since carbapenem antibiotic agents are ineffective against them [12].

Two primary mechanisms account for carbapenem resistance among *Enterobacterales*: the first mechanism involves a reduction in membrane permeability by porin loss, often associated with the production of ESBL or AmpC-β-lactamase. The second mechanism entails the production of carbapenemase enzymes capable of hydrolyzing carbapenems [13]. However, although carbapenemase and AmpC-β-lactamase production is not routinely identified in clinical microbiology laboratories in LMICs, several recent studies across African hospitals have reported carbapenemase production among *Enterobacterales* [14,15,16,17,18,19,20]. Carbapenem resistance at rates of approximately 45% has been reported among *E. coli* and *Klebsiella pneumoniae* isolates from clinical specimens in two tertiary hospitals in Nigeria [15]. A phenotypic resistance rate of 23.3% and a genotypic resistance rate of 43.1% to carbapenem were reported in Uganda [17]. Data on AmpC-β-lactamase from Africa are also accumulating gradually. In a recent study, a high rate of AmpC-β-lactamase was reported in five referral hospitals in Khartoum, with the highest rates (49.3%) among *Acinetobacter baumannii* [21].

In Burkina Faso, data on the prevalence of CRE or AmpC-β-lactamase production among clinical isolates are limited. One study conducted in a referral teaching hospital identified 17 carbapenemase-producing strains, 4 in clinical samples and 13 in fecal carriage isolates from 443 Gram-negative bacteria [19]. Another study reported NDM genes in clinical *E. coli* isolates [22]. Recent studies in the country have discovered that carbapenemase-producing bacteria or their related genes are prevalent in the hospital waste water, implying their likely presence in clinical samples as well [23,24]. In the current study, we assessed the prevalence of carbapenemase and AmpC-β-lactamase production among ESBL-producing *E. coli* (ESBL-Ec) and ESBL-producing *Klebsiella* spp. (ESBL-K) isolated from clinical samples and their co-resistance to non-betalactam antibiotics and distribution in primary, secondary and tertiary healthcare institutions in Burkina Faso.

## 2. Results

### 2.1. Bacterial Strains

A total of 473 clinical ESBL-PE isolates, comprising 356 ESBL-Ec and 117 ESBL-K, were collected from inpatients and outpatients at the five hospitals participating in the study: CHU-YO: (Centre Hospitalier Universitaire Yalgado Ouedraogo), CHR-KDG (Reginal Hospital of Koudougou), El Fateh Suka medical clinic, CMA Source de vie and CMA Saint Camille de Nanoro (Table 1).

### 2.2. Prevalence of Carbapenemase-Producing Enterobacterales

In this study, the prevalence of carbapenemase production among the 473 ESBL-PE isolates was 5.3%. The 30 meropenem-resistant isolates were tested for carbapenemase production; 25 of them, 19 (76%) *E. coli* and 6 (24%) *Klebsiella* spp., were found to produce carbapenemases (Table 1). Carbapenemase-producing isolates were identified across all the three levels of the healthcare system. The frequency was the highest among ESBL-PE isolates collected at the tertiary hospitals, with a prevalence as high as 7.5% (22/293), yet the differences did not reach statistical significance. Regarding the distribution of carbapenemase-producing isolates by sample type, the rates appeared highest in urine samples with a prevalence of 6.7% (20/299 ESBL-PE), but again, the difference was not statistically significant (Table 1). The rates among ESBL-Ec and -K were similar (5.3% and 5.2%, respectively) (Table 1).

### 2.3. Types of Carbapenemase Produced

Among the carbapenamase-producing isolates, three types of carbapenemases were detected, with NDM (19/25; 76%) as the most frequent type, followed by OXA-48-like (3/25; 12%) and VIM (1/25; 4%). Two ESBL-Ec isolates were found to produce both NDM and OXA-48-like (2/25) carbapenemases. The rates of NDM were similar among ESBL-Ec (3.9%) and ESBL-K (4.3%) isolates (Table 2).

### 2.4. Prevalence and Distribution of AmpC-β-Lactamase Production

Among the 92 presumptive AmpC-β-lactamase-producing bacterial isolates (73 ESBL-Ec and 19 ESBL-K), 25 isolates (17 ESBL-Ec and 8 ESBL-K) tested positive. The prevalence of AmpC-β-lactamase production among the ESBL-producing isolates was 5.3% (25/473), 6.8% among ESBL-K isolates and 4.8% among ESBL-Ec isolates. The prevalence of AmpC-β-lactamase-producing isolates varied from 4.7 to less than 10% in all sample types (4.7% of ESBL-PE isolates in urine samples, 5.6% in pus and 9.7% in blood cultures). The frequency among ESBL-PE in the CHU-YO hospital was 6.5% (19/293) (Table 3).

### 2.5. Antimicrobial Resistance Patterns

As the study explored ESBL-PE, the strains are, by definition, resistant to most penicillin and cephalosporins. We explored the sensitivity to piperacillin + tazobactam and found 68% of ESBL-Ec and 77% ESBL-K as resistant.

As for carbapenem resistance, which was evaluated with disc diffusion test, approximately 6% of all isolates displayed resistance to imipenem and meropenem and up to 19% of ESBL-Ec and 13% of ESBL-K to ertapenem.

The resistance rates proved high also regarding fluoroquinolones (94%) and SXT (90%) (Table 4).

In the aminoglycoside family, isolates showed high resistance rates to kanamycin (approximately 86%) and tobramycin (approximately 60%). The lowest resistance rates were recorded for amikacin: 18% among ESBL-Ec and 9% among ESBL-K isolates.

Less than 25% of all isolates were resistant to chloramphenicol. A total of 6% of ESBL-Ec and 49% of ESBL-K isolates were resistant to fosfomycin (Table 4).

## 3. Discussion

Carbapenemase and AmpC-β-lactamase detection is not routine practice in microbiology laboratories in Africa, despite several studies reporting the presence of carbapenemase- and AmpC-β-lactamase-producing *Enterobacterales* [15,21,25,26,27,28,29,30]. In our study, the prevalence of carbapenemase production was 5.3%. Twenty-five isolates, primarily *E. coli* (76%), were carbapenemase producers, most of them originating in urine samples. Our results fall within the prevalence range of 2.6–6.7% reported in North Africa, but remain somewhat lower than the range of 9.0–60.0% reported in sub-Saharan Africa in a review from 2015 [31]. In our own country, Burkina Faso, a study conducted at Sanou Sourou teaching hospital in Bobo-Dioulasso reported a much lower prevalence than the present study; they only reported rates of 0.9% for carbapenemase producers among Gram-negative bacteria in clinical specimens, most of them urine samples [19]. Another study from Burkina Faso, consistent with our study, reported NDM, VIM, OXA-48 and KPC genes among *E. coli* isolates from clinical patient samples [22]. In contrast, a study conducted in a nearby region in northwestern Nigeria described much higher carbapenemase rates than in our study: two tertiary hospitals reported a prevalence of 39% among 248 ESBL and non-ESBL *E. coli* and *Klebsiella pneumoniae* clinical isolates. These isolates were primarily obtained from urine samples [15]. These variations in prevalence could be attributed to several factors, including differences in sample types, methods employed for carbapenemase detection, and the geographical regions in which the study was conducted. Nonetheless, in all the studies, carbapenemase-producing isolates were predominantly urinary pathogens. This can be attributed to two evident reasons: first, urine samples are among the most common samples investigated in microbiological laboratories, and second, urinary tract infections constitute the most prevalent symptomatic manifestation of intestinal colonization by MDR bacteria.

Both class B (NDM and VIM) and class D (OXA-48-like) carbapenemases were detected in our study. NDM carbapenemase-producing *Enterobacterales* were the most frequent findings (76%), consistent with results of several earlier studies from sub-Saharan Africa [17,22,31,32]. In our study, we identified carbapenemase-producing isolates in three hospitals, each representing different levels of the healthcare system. CHU-YO, a referral hospital at the highest level of specialized care facilities, showed a substantial prevalence of both. This may be attributed to the more abundant use of antibiotics, the use of broader spectrum antibiotics or the prolonged duration of drug treatments, as patients are referred from medical centers, regional hospitals or medical clinics where they have already received antimicrobial treatment. Indeed, the misuse or overuse of antibiotics during hospitalization can contribute to the selection of MDR bacteria [33,34].

Production of AmpC-β-lactamases was approximately 5% among our ESBL-*E. coli* and approximately 7% among our ESBL-*Klebsiella* isolates. Production of AmpC-β-lactamases has been described for *Enterobacterales* in general in some studies in Africa at various rates [21,30], for example, at a rate of 49.3% in Sudan [21], 15.2% in Nigeria [30], 2.5% in Ethiopia [25] and 36.5% in Uganda [35]. Co-production of ESBL- and AmpC-β-lactamase at a rate of 5.2% was observed in a previous study in India [27]. In a study in Ethiopia, 3.6% of AmpC-β-lactamase-positive isolates co-produced ESBL (5/139) [25]. All these data unmistakably confirm the presence of AmpC-β-lactamase in clinical isolates. Consequently, AmpC-β-lactamase detection should also be implemented in the routines of hospitals in Africa. This is of utmost importance since AmpC-β-lactamase production in bacteria can lead to challenges and failures of treatment, with increased morbidity and mortality [30].

A total of 473 ESBL-isolates (356 *E. coli* and 117 *Klebsiella* spp.) were tested against various antibiotics. Although the resistance rates to piperacillin + tazobactam remained high, carbapenem resistance was still low.

ESBL-producing *Enterobacterales* have been investigated in numerous studies in Africa, also revealing high resistance rates to betalactamase inhibitors [18,21,36,37]. Among our ESBL-producing isolates, resistance rates to piperacillin + tazobactam appeared even higher than in many other studies; for example, in 2017–2020 in Ghana, 49% of *E. coli* and 58% of *Klebsiella* sp. in urinary samples were resistant to piperacillin + tazobactam [36], compared to 68% and 77% in our clinical samples, respectively. A recent study in Ethiopia reported much lower resistance rates (12%) to piperacillin + tazobactam among ESBL and AmpC-betalactamase-producing *Enterobacterales* [25]. The high ESBL-PE rates in LMICs and their potential co-resistance should draw the attention of clinicians who often prescribe β-lactams in prophylaxis or infection management in healthcare facilities [38].

Although carbapenem-producing *Enterobacterales* often carry both ESBL and carbapenemase genes, not all CPE strains can be covered by a study on ESBL-PE, such as the present one. However, as most of the CPE strains are, it was of interest to see in Burkina Faso what proportion of ESBL-PE are actually CPE strains. Most (76%) of the identified CPE were *E. coli*, with NDM as the most common carbapenemase type, reaching a prevalence of approximately 4% among our ESBL-PE. The respective resistance rates measured with the disc diffusion method proved somewhat higher, reaching almost 20% for ertapenem among ESBL-Ec. Similar findings were reported in previous studies across Africa; resistance rates of 0 to 14.7% to imipenem and meropenem have been reported in recent studies in Togo, Nigeria, Ethiopia and Sudan among ESBL or AmpC-β-lactamase-producing isolates [21,25,39,40]. Our results were lower compared to the 58.6% resistance against imipenem reported in India, where antibiotic consumption is higher than in Burkina Faso [41] where their use is more controlled and the drugs are very expensive. Earlier studies in Burkina Faso have reported the presence of carbapenem-resistant strains both in feces and clinical isolates [19], while in a study in Ghana [42], all ESBL isolates were susceptible to meropenem. In Burkina Faso, the presence of resistance to carbapenem could be attributed to the co-presence of genetic determinants of resistance to carbapenems with those of other antibiotics commonly used in our hospitals [23].

Among our ESBL-PE isolates, co-resistance was recorded particularly against aminoglycosides, fluoroquinolones and sulfonamides. Among aminoglycosides, high resistance rates were recorded against all regimen tested, except amikacin. This finding is in line with those reported in similar studies in Burkina Faso [19], Togo [40], Sudan [21], Ethiopia [25], Algeria [18] and India [29,43]. Interestingly, our resistance rates appear high with respect to similar studies, which show wide variations in resistance level [39,44]. Our high rates could be associated with misuse or overuse of antibiotics in hospitals, communities or farms since these antibiotics are routinely used and are easy accessed [42].

This study had several limitations. Firstly, despite collecting samples from several laboratories, it would have benefitted from a higher sample size. Secondly, this study relied on phenotypic methods. These methods primarily detect enzyme production (ESBL, AmpC-betalactamase and carbapenemases), offering information on gene activity. Genotypic detection of genes encoding these phenotypes could have revealed even higher prevalence rates. In practice, however, phenotypic methods are more feasible for hospitals in resource-limited countries and should be integrated into routine activities of microbiology laboratories across West African countries.

Amikacin, fosfomycin and chloramphenicol emerged as the most effective antibiotics in vitro. Our results align with previous research in Burkina Faso in 2021 [19], Algeria in 2019 [18], and Nigeria in 2014 [39], yet contrast the higher resistance to amikacin observed in Ghana in 2013 [44]. Apart from fosfomycin being one of the alternative drugs for cystitis (IDSA guideline), these three antibiotics are initially not preferred for many infections but rather represent reserve antibiotics in current practice. Indeed, when treating infections caused by ESBL- or carbapenemase-producing *Enterobacterales*, it is often necessary to resort to less effective reserve antibiotics.

## 4. Material and Methods

### 4.1. Study Design and Period

The prospective study was carried out in five hospitals in Burkina Faso from January 2020 to June 2022. ESBL-producing *E. coli* and *Klebsiella* spp. strains isolated from urine, blood and pus samples were collected from each hospital over a 12-month period. All isolates were characterized in the CRUN microbiology laboratory.

### 4.2. Sampling and Site Description

Burkina Faso is a West African country with a population estimated at 22,489,126 [45] and a GPT of USD 2682 per capita [46]. The health system in Burkina Faso comprises three levels. The first level encompasses peripheral healthcare facilities and primary hospitals. The second level comprises regional hospitals and certain medical clinics, which serve as reference facilities for primary hospitals. The third level includes national and teaching hospitals, representing the highest level of referral care and offering specialized services [47]. Routine practices do not cover microbiological analyses of resistant bacteria; microbiological laboratories are scarce and analyses with cultures and resistance profiles are not routinely conducted in the country.

Sampling was carried out in five hospitals, each selected to represent different levels of the health system: (1) Yalgado Ouédraogo teaching hospital (CHU-YO), a tertiary hospital located in the capital city, Ouagadougou; (2) the regional hospital of Koudougou (CHR-KDG) and (3) the El Fateh Suka medical clinic in Ouagadougou, both categorized as secondary hospitals; and two medical centers representing primary healthcare, (4) CMA Saint Camille de Nanoro, a rural medical center, and (5) CMA évangélique Source de vie in Ouagadougou. This diverse selection of healthcare units allowed a comprehensive sampling approach. A total of 473 clinical isolates were collected, comprising 356 ESBL-Ec and 117 ESBL-K strains. These isolates were collected from various clinical specimens, including urine (N = 303), pus (N = 140) and blood cultures (N = 30). The isolates were collected in tryptic soy agar tubes and kept at room temperature until transferal to the Clinical Research Unit of Nanoro (CRUN) microbiology laboratory for analysis.

### 4.3. Bacterial Isolation and Species Identification

At the CRUN microbiology lab, isolates from the tryptic soy agar tubes were plated on ESBL-selective chromogenic culture media (CHROMagar^TM^ ESBL, Paris, France). All isolates were subjected to species identification of the isolates by API 20E (Biomérieux, Marcy-l’étoile, France) according to the manufacturer’s instructions.

### 4.4. ESBL Production Test

All isolates identified were tested for ESBL production using the double disc synergy test between 3rd generation cephalosporins (ceftriaxone and ceftazidime) and 4th generation cephalosporin (cefepime) discs and amoxicillin-clavulanic acid discs according to the American Clinical and Laboratory Standards Institute (CLSI) 2022 guidelines. ESBL production was indicated by the presence of a synergistic inhibition zone between ceftazidime, ceftriaxone and/or cefepime and the amoxicillin-clavulanic acid disc.

### 4.5. Carbapenemase Production Test

Isolates were screened to select meropenem resistance for a carbapenemases production test according to the CLSI 2022 guidelines. A total of 30 isolates (22 *E. coli* and 8 *Klebsiella* spp.) that had a meropenem inhibition zone diameter less than 22 mm in the antimicrobial susceptibility test (AST) were investigated for production of the five main carbapenemases (OXA48-like, NDM, KPC, VIM and IMP) using an immunochromatographic test O.K.N.V.I. RESIST-5 (CORIS BioConcept, Gembloux, Belgium), according to the manufacturer’s instructions.

### 4.6. AmpC-β-Lactamase Production

Bacterial isolates with a cefoxitin inhibition zone diameter less than 18 mm (≤18 mm) were presumed as AmpC-β-lactamase producers. A total of 92 presumptive AmpC-β-lactamase producers’ bacterial isolates (73 ESBL-*E. coli* and 19 ESBL-*Klebsiella* spp.) were tested for AmpC-β-lactamase production using MH agar supplemented with cloxacillin at 4 µg/L. A bacterial suspension prepared with fresh colonies (McFarland 0.5) was inoculated on to the entire surface of the MH agar supplemented with cloxacillin at 4 µg/L and a disc of cefoxitin was placed on the plate. The test was positive if the inhibition zone diameter around the cefoxitin disc was ≥18 mm.

### 4.7. Antimicrobial Susceptibility Testing

An antimicrobial susceptibility test (AST) was performed using the disc diffusion method on Mueller–Hinton (MH) agar as described by Bauer et al. [48]. In total, 473 ESBL-producing isolates (356 *E. coli* and 117 *Klebsiella* spp.) were tested. The results were interpreted according to the American Clinical and Laboratory Standards Institute (CLSI) 2022 guidelines. A total of 13 antibiotics were tested, as listed in Table 4.

### 4.8. Statistical Analysis

The proportions were compared using Pearson’s Chi-square test or Fisher’s exact test as applicable.

## 5. Conclusions

Our study in Burkina Faso revealed that approximately 5% of ESBL-producing *E. coli* and *Klebsiella* spp. isolates produced carbapenemases and/or AmpC-βlactamases. These highly resistant pathogens were found at all levels of healthcare. Three types of carbapenemases, NDM, OXA-48-like and VIM, were detected, with NDM as the most prevalent. The antibiotic resistance patterns revealed notable co-resistance to antibiotics commonly used for patient treatment. Implementing antibiotic stewardship in all levels of healthcare and establishing effective and reliable AMR surveillance systems are essential measures for containing resistant bacteria and preventing their dissemination within hospitals and into the broader communities.

## Figures and Tables

**Table 1:** Prevalence of carbapenemase-producing isolates among extended-spectrum betalactamase (ESBL)-producing *Enterobacterales*: data provided by hospitals and sample type.

	ESBL- *E. coli* N = 356 n/N (%)	ESBL- *Klebsiella* spp. N = 117 n/N (%)	All Isolates N = 473 n/N (%)	Prevalence (%)	*p*-Value
**Hospitals**					0.14 ^a^
CHU-YO	18/211 (8.5)	4/82 (4.9)	22/293	7.5	
CHR-KDG	1/44 (2.3)	0/13 (0)	1/57	1.8	
El Fateh Suka medical clinic	0/24 (0)	0/0 (0)	0/24	0	
CMA Saint Camille de Nanoro	0/58 (0)	2/14 (1.4)	2/74	2.7	
CMA évangélique source de vie	0 /19 (0)	0/8 (0)	0/27	0	
**Sample Type**					0.20 ^b^
Urine	16/228 (7.0)	4/71 (5.6)	20/299	6.7	
Pus	2/113 (1.8)	2/30 (6.7)	4/143	2.8	
Blood culture	1/15 (6.7)	0/16 (0)	1/31	3.2	
**Overall prevalence**	19/356 (5.3)	6 /117(5.1)	25/473	5.3	

N = number of isolates tested; n = number of resistant isolates; ^a^ Fisher’s exact test for count data for comparisons between healthcare settings. ^b^ Pearson’s Chi-squared test for comparisons between sample types.

**Table 2:** Carbapenemase findings among ESBL-PE isolates and proportions of each carbapenemase type.

	ESBL- *E. coli* N = 356 n (%)	ESBL- *Klebsiella* spp., N = 117 n (%)	All Isolates N = 473 n (%)	Isolates with Carbapenemase N = 25 n (%)
**Carbapenemases**				
NDM	14 (3.9)	5 (4.3)	19 (4.0)	19/25 (76)
OXA-48-like	3 (0.8)	0 (0)	3 (0.6)	3/25 (12)
OXA-48-like + NDM	2 (0.6)	0 (0)	2 (0.4)	2/25 (8)
VIM	0 (0)	1 (0.9)	1 (0.2)	1/25 (4)
**Total**	19 (5.3)	6 (5.1)	25 (5.3)	25 (100)

N = number of isolates tested; n = number of resistant isolates.

**Table 3:** Prevalence of AmpC-β-lactamase-producing isolates among ESBL-producing *Enterobacterales*; data provided by hospital and type of sample.

	ESBL- *E. coli* N *=* 356 n/N (%)	ESBL- *Klebsiella* spp. N = 117 n/N (%)	All Isolates N = 473 n/N (%)	Prevalence (%)	*p*-Value
**Hospitals**					0.12 ^a^
CHU-YO	13/211 (6.2)	6 /82 (7.3)	19/293	6.5	
CHR-KDG	0/44 (0)	0/13 (0)	0/57	0	
El Fateh Suka medical clinic	2 /24 (8.3)	0/0 (0)	2/24	8.3	
CMA Saint Camille de Nanoro	1/58 (1.7)	1/14 (7.1)	2/74	2.7	
CMA évangélique Source de vie	1/19 (5.3)	1/8 (12.5)	2/27	7.4	
**Sample Type**					0.49 ^b^
Urines	8/228 (3.5)	6/71 (8.5)	14/299	4.7	
Pus	7/113 (6.2)	1/30 (3.3)	8/143	5.6	
Bloodculture	2/15 (13.3)	1/16 (6.3)	3/31	9.7	
**Overall prevalence**	17/356 (4.8)	8/117 (6.8)	25/473	5.3	

N = number of isolates tested; n = number of resistant isolates; ^a^ Fisher’s exact test for count data for comparisons between healthcare settings. ^b^ Pearson’s Chi-squared test for comparisons between sample types.

**Table 4:** Resistance to various antibiotics among ESBL-producing *E. coli* and *Klebsiella* sp.; isolates tested via the disc diffusion method.

Antibiotics (Concentration in µg)	ESBL- *E. coli* N = 356	ESBL-*Klebsiella* spp. N = 117
	Resistance	Resistance
	n (%)	n (%)
Piperacillin + Tazobactam (110)	242 (68.0)	90 (76.9)
Meropenem (10)	22 (6.2)	8 (6.8)
Imipenem (10)	21 (5.9)	8 (6.8)
Ertapenem (10)	69 (19.4)	15 (12.8)
Gentamicin (10)	153 (42.9)	68 (58.1)
Amikacin (30)	65 (18.3)	11 (9.4)
Tobramycin (10)	215 (60.4)	69 (59.0)
Kanamycin (30)	303 (85.1)	101 (86.3)
Ciprofloxacin (5)	335 (94.1)	110 (94.0)
Sulfamethoxazole + trimethoprim (25)	297 (834)	105 (89.7)
Nitrofurantoin (300)	118 (33.1)	87 (74.4)
Fosfomycin (200)	20 (5.6)	57 (48.7)
Chloramphenicol	71 (19.9)	29 (24.8)

N = number of isolates tested; n = number of resistant isolates.

## Data Availability

Data are contained within the article.
